# Modelling the complexity of pandemic-related lifestyle quality change and mental health: an analysis of a nationally representative UK general population sample

**DOI:** 10.1007/s00127-021-02210-w

**Published:** 2021-12-16

**Authors:** Sarah Butter, Jamie Murphy, Philip Hyland, Orla McBride, Mark Shevlin, Todd K. Hartman, Kate Bennett, Jilly Gibson-Miller, Liat Levita, Anton P. Martinez, Liam Mason, Ryan McKay, Thomas V. A. Stocks, Frédérique Vallières, Richard P. Bentall

**Affiliations:** 1 Department of Psychology, University of Sheffield, Cathedral Court, 1 Vicar Lane, Sheffield, S1 2LT England; 2 School of Psychology, Ulster University, Coleraine, Northern Ireland; 3 Department of Psychology, Maynooth University, Kildare, Ireland; 4 Sheffield Methods Institute, University of Sheffield, Sheffield, England; 5 Department of Psychology, University of Liverpool, Liverpool, England; 6 Division of Psychology and Language Sciences, University College London, London, England; 7 Department of Psychology, Royal Holloway, University of London, London, England; 8 Trinity Centre for Global Health, Trinity College Dublin, Dublin, Ireland

**Keywords:** Lifestyle, COVID-19, Pandemic, Mental health, Relationships, Latent variable modelling, UK

## Abstract

**Purpose:**

The COVID-19 pandemic has affected the way many individuals go about their daily lives. This study attempted to model the complexity of change in lifestyle quality as a result of the COVID-19 pandemic and its context within the UK adult population.

**Methods:**

Data from the COVID-19 Psychological Research Consortium Study (Wave 3, July 2020; *N* = 1166) were utilised. A measure of COVID-19-related lifestyle change captured how individuals’ lifestyle quality had been altered as a consequence of the pandemic. Exploratory factor analysis and latent profile analysis were used to identify distinct lifestyle quality change subgroups, while multinomial logistic regression analysis was employed to describe class membership.

**Results:**

Five lifestyle dimensions, reflecting partner relationships, health, family and friend relations, personal and social activities, and work life, were identified by the EFA, and seven classes characterised by distinct patterns of change across these dimensions emerged from the LPA: (1) better overall (3.3%), (2) worse except partner relations (6.0%), (3) worse overall (2.5%), (4) better relationships (9.5%), (5) better except partner relations (4.3%), (6) no different (67.9%), and (7) worse partner relations only (6.5%). Predictor variables differentiated membership of classes. Notably, classes 3 and 7 were associated with poorer mental health (COVID-19 related PTSD and suicidal ideation).

**Conclusions:**

Four months into the pandemic, most individuals’ lifestyle quality remained largely unaffected by the crisis. Concerningly however, a substantial minority (15%) experienced worsened lifestyles compared to before the pandemic. In particular, a pronounced deterioration in partner relations seemed to constitute the more severe pandemic-related lifestyle change.

**Supplementary Information:**

The online version contains supplementary material available at 10.1007/s00127-021-02210-w.

## Introduction

The strict COVID-19-related restrictions and regulations (e.g. lockdowns, social distancing) that have been put in place have considerably changed many individuals’ daily lives, routines, and relationships [1]. These measures have been effective in suppressing the transmission of the disease and will likely remain in place pending full rollout of vaccines that have been developed [2]. As a result, there has been widespread and ongoing concern that government-prescribed mitigation measures have, alongside virus-related morbidity, mortality and grief, negatively impacted the mental health of the general population [1, 3–5]. A recent meta-analysis examining changes in mental health pre- and post-COVID-19 outbreak suggests that there has been a small overall increase in symptoms of anxiety and depression [6]. However, psychological responses since the outbreak appear to be highly heterogeneous [7, 8].

Specific COVID-19-related lifestyle changes have been documented in relation to health behaviours, including decreased physical activity, increased sedentary behaviour, screen time, food and alcohol consumption, unhealthier food choices, and poorer sleep [9–11]. Moreover, disruptions to personal relationships and social activity have been common [12–16]. Many individuals may also be under increased financial pressures as a result of unemployment or reduced working hours [17–19], while employed individuals may be struggling with changes to work schedules, roles and adjusting to a home-working environment [15, 20–22]. These negative lifestyle changes have been associated with poor mental health outcomes during this crisis in a number of studies [10, 22, 23]. Yet, it is underappreciated that individuals may also respond positively to the changes in life brought about by the COVID-19 pandemic. For example, some studies have identified groups of individuals who are living healthier lifestyles during the pandemic [9], reported improvements in their relationship satisfaction [16], and have decreased their overall spending [19, 24], all of which may positively impact their lives.

Importantly, however, these changes do not affect everyone equally. For example, unemployment and financial difficulties appear to be affecting the young, least educated, ethnic minorities, women and those with pre-existing financial difficulties the most [17, 19, 25]. Parents are negatively affected by school closures, particularly mothers who appear to be shouldering more caring responsibilities and are more likely to experience negative employment outcomes as a result of home-schooling demands [19, 26, 27]. Additionally, those living in cramped or poor housing conditions may also be more negatively affected [28]. Furthermore, particular lifestyle changes may be likely to co-occur with one another. For example, financial strain experienced by couples during the pandemic may negatively affect their relationship quality and satisfaction [29, 30].

To model and describe the complexity of pandemic-related lifestyle quality change, as well as its context and potential consequences, the following study had three objectives. First, using a broad array of social, health, relationship and economic lifestyle indicators that were designed to capture pandemic-induced lifestyle changes, we sought to identify the primary dimensions of lifestyle quality change within the general adult population during the first 4 months of the COVID-19 pandemic. In doing so, we aimed to distil the complexity of these many and varied lifestyle changes into a more parsimonious representation of pandemic-related change in lifestyle quality. Second, we then sought to identify if there were distinct groups within the population who were characterised by the same profile of variation across the dimensions of lifestyle quality change. This afforded an opportunity to identify, not only, what aspects of lifestyle changed for distinct groups within the population, but whether the lifestyles of these groups were changing for better or worse. Third, we aimed to describe and differentiate the membership of the distinct lifestyle quality change groups using a variety of sociodemographic, COVID-19-related, psychological, and mental health variables to better understand whose lives were changing and potentially reveal some of the factors that may be underlying these changes.

## Method

### Sample

Launched in March 2020, the COVID-19 Psychological Research Consortium (C19PRC) Study is an online, longitudinal study which was designed to measure, assess, and monitor the population’s psychological and social adjustment to the COVID-19 pandemic. Briefly, at baseline (referred to as C19PRC-UKW1; 23–28 March 2020), 2025 adults aged 18 years and older were recruited via the survey company *Qualtrics*. The current study utilised follow-up survey data collected from this cohort during 9–23 July 2020, Wave 3 of the study (C19PRC-UKW3; *N* = 1166; 57.6% retention rate), approximately 4 months after the first nationwide lockdown was imposed in the UK. During this point in the pandemic, the UK was at the tail end of its first wave of COVID-19 when the number of daily new confirmed positive COVID-19 cases and daily death rate had declined considerably from peaks in mid-to-late April 2020 (see [31] for details).

Quota sampling methods were used at baseline to ensure the sample was representative of the UK adult population in terms of age, sex, and gross household income. Additionally, the baseline sample was broadly in line with population-level estimates in relation to economic activity, ethnicity, household composition and residency within the UK. Post-stratification weights were applied to the Wave 3 sample to ensure it was as representative of the baseline as possible. Thus, the current sample mirrored the population distribution of the baseline sample regarding age, sex, income, ethnicity, urbanicity, household composition and being born or raised in the UK. Ethical approval for the study was granted by the University of Sheffield (Ref no. 033759). A detailed methodological account of the C19PRC-UK Study, including sampling and design, fieldwork procedures, quality control, sample representativeness, weighting and attrition at Wave 1–Wave 3 can be found elsewhere [31, 32].

### Measures

#### Pandemic-related lifestyle change

A series of questions were generated for the Wave 3 study to assess changes to respondents’ lifestyle quality as a result of the pandemic [31]. Respondents were presented with a list of 19 lifestyle indicators including relationship with intimate partner, work role, exercise, and religious/spiritual life, and asked to indicate whether their life was ‘Better’ (1), ‘No different’ (2) or ‘Worse’ (3) now than before the COVID-19 pandemic in relation to each indicator. Respondents were also presented with a ‘Not applicable’ response (e.g. non-religious individuals could endorse this option for the item regarding changes in their ‘religious/spiritual life’); for the purposes of the current study, this response was collapsed into the ‘No different’ category to indicate lack of change since the pandemic began. The 19 lifestyle indicators used in the study are presented in Table 1.

**Table 1:** Endorsement of pandemic-related lifestyle change (*N* = 1166, weighted data)

Please indicate if the following areas of your life are better or worse now than they were before the COVID-19 pandemic?	*N* (%)			
	No different	Better	Worse	Ratio worse:better
Home life	791 (67.8)	200 (17.2)	175 (15.0)	0.9:1
Relationship with your intimate partner	892 (76.5)	157 (13.4)	118 (10.1)	0.8:1
Relationship with your family	873 (74.9)	183 (15.7)	110 (9.5)	0.6:1
Relationship with your children	947 (81.2)	152 (13.1)	67 (5.7)	0.4:1
Relationship with your friends	878 (75.3)	94 (8.1)	194 (16.7)	2.1:1
Diet	664 (57.0)	181 (15.5)	321 (27.5)	1.8:1
Exercise	538 (46.1)	280 (24.0)	349 (29.9)	1.2:1
Taking care of your mental health	773 (66.3)	115 (9.8)	278 (23.9)	2.4:1
Taking care of your physical health	665 (57.1)	214 (18.4)	286 (24.6)	1.3:1
Work–life balance	783 (67.1%)	214 (18.3)	170 (14.5)	0.8:1
Work role	866 (74.3)	107 (9.2)	193 (16.5)	1.8:1
Relationship with your work colleagues	951 (81.6)	92 (7.9)	123 (10.6)	1.3:1
Time spent commuting	792 (67.9)	314 (26.9)	60 (5.1)	0.2:1
Education/personal development	933 (80.0)	127 (10.9)	106 (9.1)	0.8:1
Socialising	565 (48.5)	75 (6.4)	526 (45.1)	7:1
Sex life	914 (78.4)	82 (7.1)	170 (14.6)	2.1:1
Engagement in hobbies and pastimes	716 (61.4)	225 (19.3)	225 (19.3)	1:1
Religious or spiritual life	1012 (86.8)	56 (4.8)	98 (8.4)	1.8:1
Social media use	877 (75.2)	145 (12.4)	144 (12.4)	1:1

#### Predictor variables

A number of sociodemographic, COVID-19-related, psychological and mental health variables were used to predict patterns of pandemic-related lifestyle quality change. Full details of predictor variables are available in Online Resource 1.

Sociodemographic variables: age, gender (male, female), ethnicity (white British/Irish, ethnic minority), urbanicity (city, suburb/town/rural), current economic activity (active, inactive), gross household income (pre-pandemic, in 2019; £0–£300 per week, £301–£490 per week, £491–£740 per week, £641–£1,111 per week, £1,112 + per week), relationship status (in a relationship, not in a relationship), presence of dependent children in the home (yes, no), and number of bedrooms in the household (0/1 through to 5 +). All sociodemographic variables were measured at Wave 3 (July 2020) with the exception of urbanicity, ethnicity and gross household income (measured at Wave 1; March 2020).

COVID-19-related variables: respondents were also asked whether they were a government-defined keyworker (yes, no), living in an area under local lockdown at the time of the study (yes, no), whether their monthly household income had changed since before the pandemic (lost income, no lost income), and whether they had a chronic health condition (self and close family members; yes, no), as well as about their degree of COVID-19 related anxiety (low, medium, high), perceived COVID-19 infection status (self and family members; infected, not infected), and if someone close to them had died from COVID-19 (yes, no). All COVID-19-related variables were measured at Wave 3 with the exception of self and family member health conditions, which were measured at Wave 1.

Psychological related variables: several psychological variables measured at Wave 3 were included: loneliness was measured by the three-item Loneliness Scale [33], happiness with the Subjective Happiness Scale [34], hopefulness with the Brief-H-Pos Scale [35] and social support with the Modified Medical Outcome Social Support Survey (mMOS-SSS) [36]. Additional psychological scales, measured at Wave 1 were also included: resilience was measured using the Brief Resilience Scale [37], death anxiety with Death Anxiety Inventory [38], and intolerance of uncertainty with Intolerance of Uncertainty scale [39].

Mental health variables: several variables measured at Wave 3 were included as predictor variables. Depression was measured using the Patient Health Questionnaire-9 (PHQ-9) [40], generalised anxiety with the Generalized Anxiety Disorder 7-item Scale (GAD-7) [41], and COVID-19-related posttraumatic stress with a modified version of the International Trauma Questionnaire (ITQ) [42]. Additionally, suicidal ideation since the outbreak of the pandemic was also measured in the sample.

### Analytic plan

To address objectives 1 and 2, latent variable modelling was conducted in two stages. First, exploratory factor analysis (EFA; maximum likelihood extraction, oblique rotation) was employed to determine the latent structure of change in lifestyle quality during the pandemic based on responses to the 19 items measuring lifestyle change. The number of factors to be extracted was determined using eigenvalues as well as the interpretability and meaningfulness of the solution. Second, after establishing the appropriate number of dimensions of lifestyle quality change using EFA, latent profile analysis (LPA) was used to identify homogeneous groups, or classes, based on scores of these dimensions of lifestyle quality change. The fit of seven models (a two-class through an eight-class model) was assessed using LPA, with the factor scores generated from the EFA as continuous indicators.

The relative fit of the LPA models was compared by using three information theory-based fit statistics: the Akaike information criterion (AIC) [43], the Bayesian information criterion (BIC) [44] and the sample size-adjusted Bayesian information criterion (ssa-BIC) [45]. The model that produced the lowest values was judged to be the best-fitting model. However, the BIC is considered to be the best of the fit indices for deciding the number of classes in LPA [46]. The Lo–Mendell–Rubin likelihood ratio test (LRT) [47] can also be used to determine class enumeration. When the LRT becomes non-significant, it suggests the model with one less class is a better fit to the data. In addition to the fit statistics, it is important to consider the conceptual relevance of the latent profiles when interpreting the results. Robust maximum likelihood estimation [48] was used for the LPAs. To avoid solutions based on local maxima, 100 random sets of starting values were initially used, with 10 final stage optimisations.

To address objective 3, multinomial logistic regression was carried out to assess whether sociodemographic, COVID-19-specific, psychological and mental health variables could discriminate between class membership of the best fitting LPA. Analyses were conducted using SPSS v26 and Mplus version 7 [49]. Weighting procedures (see ‘Sample’) were applied when conducting the descriptive, EFA, and regression analyses.

## Results

Endorsement frequencies for the 19 lifestyle quality indicators are reported in Table 1. More than half of respondents (57.0–86.8%) indicated ‘no difference’ in each area of their life compared to before the pandemic, with the exception of ‘Exercise’ and ‘Socialising’. In relation to ‘Exercise’, a quarter of individuals (24.0%) indicated that this had improved, while three in ten reported that it had deteriorated. Additionally, almost half of the sample (45.1%) indicated that their ‘Socialising’ was worse now than before the pandemic, whereas only around 1 in 15 (6.4%) reported that it had improved. Other than ‘Socialising’, variables which had the largest worse:better ratios included ‘taking care of your mental health’ (2.4:1), ‘relationship with your friends’ (2.1:1), and ‘sex life’ (2.1:1), while ‘relationship with your children’ (0.4:1) and ‘time spent commuting’ (0.2:1) had the smallest worse:better ratios.

### Exploratory factor analysis

Data suitability was assessed prior to conducting EFA. These results suggested that singularity was not a problem (determinant = 0.010), and that the sample and correlation matrix were factorable (KMO = 0.84; Bartlett’s Test of Sphericity: *χ*^2^ = 5348.27, df = 171, *p* < 0.001). Correlations ranged between 0.06 and 0.68 (see Online Resource 1). Six dimensions were initially extracted with eigenvalues greater than one; however, the scree plot suggested that five or six dimensions might be retained. Further inspection of the six-factor solution revealed that it contained a dimension on which only one item loaded above 0.30; therefore, this model was dismissed as a viable solution. Further inspection of the five-factor solution revealed dimensions which were conceptually distinguishable. The results of maximum likelihood extraction with oblique (direct oblimin) rotation of this solution are presented in Table 2.

**Table 2:** Factor analysis of lifestyle items (*N* = 1166, weighted data)

Lifestyle items	Factor				
	1	2	3	4	5
Home life	**0.369**	0.098	0.195	0.228	0.004
Relationship with your intimate partner	**1.025**	− 0.020	− 0.002	0.013	− 0.077
Relationship with your family	− 0.020	0.006	− 0.011	**0.786**	0.000
Relationship with your children	0.097	− 0.002	− 0.011	**0.500**	− 0.008
Relationship with your friends	− 0.065	0.057	0.131	**0.257**	0.200
Diet	− 0.014	**0.544**	0.087	− 0.004	− 0.009
Exercise	0.025	**0.812**	− 0.096	0.010	− 0.034
Taking care of your mental health	0.108	**0.397**	0.124	− 0.069	0.203
Taking care of your physical health	− 0.018	**0.862**	− 0.006	0.052	− 0.014
Work–life balance	0.015	0.059	**0.566**	0.052	0.042
Work role	− 0.003	0.035	**0.828**	0.006	− 0.130
Relationship with your work colleagues	0.043	− 0.016	**0.480**	− 0.037	0.144
Time spent commuting	0.056	− 0.036	**0.246**	0.036	0.190
Education/personal development	0.078	− 0.035	0.086	− 0.088	**0.664**
Socialising	− 0.011	0.133	0.086	− 0.037	**0.275**
Sex life	**0.350**	0.057	− 0.007	0.048	0.223
Engagement in hobbies and pastimes	0.040	0.095	− 0.046	0.115	**0.503**
Religious or spiritual life	0.014	0.039	− 0.054	0.041	**0.528**
Social media use	− 0.010	− 0.045	0.049	0.048	**0.320**
Eigenvalue	4.909	1.654	1.471	1.185	1.046
%Variance explained	25.84%	8.71%	7.74%	6.24%	5.51%
Factor correlations					
F1: partner relationship	–				
F2: healthy lifestyle	0.285	–			
F3: work life	0.312	0.362	–		
F4: family and friends	0.407	0.260	0.251	–	
F5: personal and social activities	0.359	0.369	0.553	0.371	–

Strongest loading for each item in bold. Extraction: maximum likelihood; rotation: oblimin. Total variance explained: 54.03%

Three items loaded onto factor 1: relationship with intimate partner, home life, and sex life. This was labelled as the ‘Partner relationship’ dimension. Four items loaded onto factor 2 (taking care of your physical health, exercise, diet and taking care of your mental health), clearly reflecting a ‘Healthy lifestyle’ dimension. Four items loaded onto factor 3: work role, work-life balance, relationship with your work colleagues and time spent commuting. This was considered to reflect a ‘Work life’ dimension. Factor 4 contained three items, relationships with your family, children, and friends; this dimension was labelled ‘Family and friends’. Finally, five items loaded onto factor 5: education/personal development, religious/spiritual life, engagement in hobbies and pastimes, social media use and socialising. This factor was labelled as the ‘Personal and social activities’ dimension. All items loaded positively onto their respective dimensions, indicating that higher scores related to worsening of that aspect of lifestyle. The strongest factor correlations were between ‘Personal and social activities’ and ‘Work life’ (*r* = 0.55) and between ‘Partner relationship’ and ‘Family and friends’ (*r* = 0.41).

Three indicators had low factor loadings (< 0.30). These were ‘Relationship with your friends’ (0.257), ‘Time spent commuting’ (0.246) and ‘Socialising’ (0.275). The current study sought to examine the dimensionality of lifestyle change during the pandemic, rather than test a theoretical model of lifestyle change. Therefore, these items were included in the model despite their low factor loadings. This decision was also as a result of the conceptual relevance of each of these items within their respective factors, and the large sample size used in the study [50].

### LPA on factor scores

**Fig. 1:**
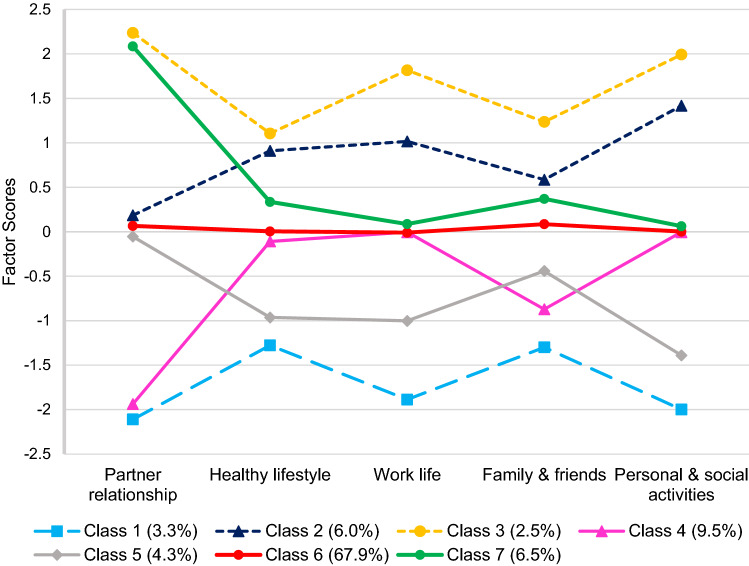
Seven-class latent profile analysis plot modelled using weighted factor scores from five-factor EFA solution. Higher scores indicate worsening on that lifestyle dimension

Fit indices for the LPAs are shown in Table 3. Class enumeration was based on both statistical and conceptual considerations. The AIC, BIC and ssaBIC continued to decrease from the two-class model through to the eight-class model. The LRT, however, became non-significant in the eight-class model. This result, combined with the decreasing BIC throughout the models, suggested that the seven-class model should be accepted. Inspection of the seven-class structure revealed the presence of distinct classes, with each class capturing a unique pattern of change or stability in lifestyle quality across the five dimensions (Fig. 1). Additionally, this solution indicated acceptable classification of participants (entropy = 0.98).

**Table 3:** Fit statistics for latent profile analysis of weighted factor scores (*N* = 1166)

Classes	Log-likelihood	AIC	BIC	ssaBIC	Entropy	LRT, *p*
2	− 6950.107	13,932.213	14,013.195	13,962.373	0.959	865.300, *p* < 0.001
3	− 4707.852	9459.705	9571.054	9501.175	1.000	4381.104, *p* < 0.001
4	− 4443.349	8942.698	9084.415	8995.477	0.994	516.809, *p* < 0.001
5	− 4162.936	8393.871	8565.957	8457.961	0.971	547.894, *p* < 0.01
6	− 3902.069	7884.137	8086.591	7959.537	0.974	509.705, *p* < 0.01
**7**	− **3609.170**	**7310.339**	**7543.161**	**7397.049**	**0.978**	**572.290,** ***p* < 0.05**
8	− 3485.410	7074.820	7338.009	7172.839	0.976	241.812 *p* > 0.05

Selected model in bold

*AIC* Akaike information criterion, *BIC* Bayesian information criterion, *ssaBIC* sample size-adjusted BIC, *LRT* Lo–Mendell–Rubin adjusted likelihood ratio test

Class 1 (Better overall lifestyle; *n* = 38, 3.3%) comprised a group of adults whose factor scores indicated improvement across all dimensions (i.e. below 0 across all factors). Class 2 (Worse except partner; *n* = 70, 6.0%) consisted of a group whose profile indicated no difference on partner relations, but lifestyle deterioration across all other dimensions. Similarly, Class 3 (Worse overall lifestyle; *n* = 29, 2.5%), was the smallest class and represented a group of adults whose factor scores indicated worsening across all lifestyle dimensions, including partner relations. Class 4 (Better relationships; *n* = 111, 9.5%) comprised a group of adults with improvements mainly to their partner relationship/home life, as well as their relationships with family and friends, but with no difference on the remaining dimensions. Class 5 (Better except partner; *n* = 50, 4.3%), was similar in profile to Class 1, with the exception that these individuals reported no difference to their partner relationships. Class 6 (No different; *n* = 792, 67.9%), was the largest subgroup, and comprised adults who reported relatively little change in their lifestyle since before the pandemic. Finally, Class 7 (Worse partner only; *n* = 76, 6.5%), consisted of a group who reported little difference to their lifestyle in relation to their engagement in healthy behaviours, work life, family/friend relationships and personal and social activities; however, their partner relationship greatly deteriorated.

### Predicting class membership

Odds ratios (ORs) for the sociodemographic, COVID-19-related, mental health and psychological variables predicting class membership are shown in Table 4. Class 6 ‘No different’ was used as the reference class. Having a better overall lifestyle now than before the pandemic (Class 1), was associated with being economically active (OR = 3.90), being in a relationship (OR = 2.95), living in a larger house (5 bedrooms compared to 3 bedrooms; OR = 7.14), and having higher levels of hopefulness (OR = 1.55) and death anxiety (OR = 1.05). The worse lifestyle except partner relationships class (Class 2) were more likely to be economically active (OR = 2.36) and have higher levels of loneliness (OR = 1.22). Having a worse overall lifestyle (Class 3) was associated with living in a city (OR = 3.57), being of white British/Irish ethnicity (OR = 7.46), being in a relationship (OR = 7.99), being less likely to have a health condition (OR = 8.33), being more likely to be a keyworker (OR = 4.76), living in a local lockdown (OR = 4.86), having higher levels of death anxiety (OR = 1.07), meeting the criteria for COVID-19 PTSD (OR = 5.88) and reporting suicidal ideation since the beginning of the pandemic (OR = 4.24).

**Table 4:** Multinomial logistic regression predicting class membership (odds ratios) (*N* = 1049, weighted data)

	*N* (%)/means (range)	OR (95% CI)					
		Class 1 (3.3%) Better overall	Class 2 (6.0%) Worse except partner	Class 3 (2.5%) Worse overall	Class 4 (9.5%) Better relationships	Class 5 (4.3%) Better except partner	Class 7 (6.5%) Worse partner only
Age	45.80 (18–87)	0.98 (0.95–1.01)	0.99 (0.97–1.02)	0.99 (0.94–1.04)	0.99 (0.97–1.01)	0.97 (0.94–0.99)**	1.01 (0.98–1.03)
Gender							
Male	565 (48.6)	0.54 (0.24–1.23)	1.19 (0.65–2.18)	1.87 (0.59–5.91)	0.84 (0.52–1.36)	0.92 (0.46–1.84)	0.62 (0.33–1.18)
Female	598 (51.4)	–	–	–	–	–	–
Urbanicity							
Suburb/town/rural	879 (75.4)	0.61 (0.27–1.41)	1.28 (0.62–2.68)	0.28 (0.08–0.95)*	0.77 (0.44–1.35)	1.62 (0.73–3.59)	1.90 (0.83–4.32)
City	287 (24.6)	–	–	–	–	–	–
Economic activity							
Economically active	765 (65.6)	3.90 (1.20–12.63)*	2.36 (1.10–5.05)*	1.13 (0.18–7.11)	1.20 (0.64–2.24)	3.26 (1.34–7.95)**	0.88 (0.38–2.04)
Not economically active	401 (34.4)	–	–	–	–	–	–
Gross income 2019							
£1,112 or more per week	236 (20.2)	0.41 (0.08–2.17)	1.90 (0.63–5.72)	0.99 (0.11–8.65)	3.17 (1.12–8.98)*	1.36 (0.44–4.23)	2.42 (0.69–8.41)
£741–£1,111 per week	236 (20.2)	0.71 (0.16–3.22)	0.68 (0.21–2.26)	0.37 (0.04–3.42)	2.07 (0.73–5.87)	1.27 (0.41–3.96)	3.18 (1.01–9.99)*
£491–£740 per week	222 (19.0)	1.92 (0.50–7.44)	1.11 (0.39–3.13)	1.01 (0.14–7.14)	2.16 (0.76–6.15)	0.84 (0.29–2.44)	3.84 (1.36–10.81)*
£301–£490 per week	236 (20.2)	1.78 (0.45–6.95)	1.40 (0.57–3.44)	0.60 (0.08–4.26)	3.17 (1.18–8.53)*	0.41 (0.13–1.31)	1.17 (0.39–3.51)
£0–£300 per week	236 (20.2)	–	–	–	–	–	–
Ethnicity							
White British/Irish	1018 (87.3)	1.08 (0.36–3.24)	2.25 (0.74–6.85)	7.46 (1.00–55.84)*	0.74 (0.36–1.52)	0.43 (0.18–1.01)	0.81 (0.35–1.87)
Ethnic minority	148 (12.7)	–	–	–	–	–	–
In a relationship							
Yes	716 (61.4)	2.95 (1.17–7.42)*	1.08 (0.53–2.21)	7.99 (1.96–32.62)**	4.75 (2.30–9.84)***	0.24 (0.11–0.55)***	5.48 (2.44–12.31)***
No	450 (38.6)	–	–	–	–	–	–
Children in the home							
Yes	328 (28.1)	0.87 (0.35–2.19)	0.74 (0.34–1.58)	2.36 (0.66–8.39)	1.45 (0.85–2.48)	1.20 (0.58–2.50)	2.75 (1.39–5.45)**
No	838 (71.9)	–	–	–	–	–	–
No. of bedrooms in the home							
Bedrooms: none/1	126 (10.8)	0.34 (0.07–1.79)	1.61 (0.30–8.67)	0.54 (0.04–7.68)	3.32 (0.92–11.93)	0.21 (0.04–1.13)	3.83 (0.21–70.17)
2	254 (21.8)	0.40 (0.09–1.79)	0.78 (0.16–3.85)	0.14 (0.01–1.66)	1.78 (0.58–5.50)	0.50 (0.13–1.86)	13.79 (1.03–184.95)*
3	486 (41.7)	0.14 (0.03–0.63)**	0.96 (0.22–4.23)	0.13 (0.01–1.17)	1.34 (0.48–3.77)	0.46 (0.14–1.54)	5.37 (0.42–68.31)
4	232 (19.9)	1.04 (0.25–4.25)	0.93 (0.20–4.40)	0.16 (0.01–1.77)	1.44 (0.50–4.17)	0.45 (0.12–1.63)	6.08 (0.47–78.92)
5	68 (5.8)	–	–	–	–	–	–
C19-related lost income							
Yes	377 (32.4)	0.78 (0.33–1.85)	1.78 (0.97–3.28)	1.53 (0.47–4.94)	1.22 (0.75–1.98)	1.99 (1.04–3.80)*	2.06 (1.10–3.84)*
No	789 (67.6)	–	–	–	–	–	–
Health condition—self							
Yes	159 (13.6)	0.71 (0.19–2.66)	0.49 (0.17–1.40)	0.12 (0.02–0.78)*	1.40 (0.66–2.95)	2.43 (0.94–6.32)	0.85 (0.34–2.15)
No	1007 (86.4)	–	–		–	–	–
Health condition—family							
Yes	281 (24.1)	0.94 (0.36–2.44)	1.89 (0.92–3.90)	2.37 (0.62–9.00)	0.48 (0.25–0.91)*	0.82 (0.36–1.90)	2.18 (1.09–4.33)*
No	885 (75.9)	**–**	**–**	**–**	**–**	**–**	**–**
C19 infection—self							
Yes	104 (8.9)	1.48 (0.50–4.38)	0.86 (0.33–2.28)	0.54 (0.10–2.97)	1.01 (0.46–2.19)	0.66 (0.17–2.54)	0.81 (0.31–2.09)
No	1062 (91.1)	**–**	**–**	**–**	**–**	**–**	**–**
C19 infection—family member							
Yes	113 (9.7)	2.00 (0.64–6.27)	1.45 (0.57–3.68)	0.55 (0.09–3.57)	1.58 (0.71–3.53)	1.18 (0.41–3.39)	1.07 (0.39–2.92)
No	1053 (90.3)	**–**	**–**	**–**	**–**	**–**	**–**
C19 death—someone close							
Yes/uncertain	70 (6.0)	1.99 (0.43–9.21)	0.97 (0.27–3.51)	2.72 (0.36–20.69)	1.30 (0.47–3.59)	1.21 (0.29–5.04)	1.65 (0.54–5.07)
No	1096 (94.0)	**–**	**–**	**–**	**–**	**–**	**–**
C19 anxiety							
High	359 (30.8)	1.55 (0.57–4.25)	0.62 (0.27–1.43)	2.58 (0.49–13.55)	1.13 (0.62–2.08)	1.74 (0.74–4.10)	2.42 (0.99–5.96)
Moderate	475 (40.8)	1.68 (0.65–4.37)	1.10 (0.53–2.25)	2.24 (0.46–10.78)	1.04 (0.60–1.80)	0.82 (0.36–1.87)	1.97 (0.83–4.68)
Low	331 (28.4)	**–**	**–**	**–**	**–**	**–**	**–**
Keyworker							
No	854 (73.2)	1.40 (0.58–3.34)	1.73 (0.81–3.68)	0.21 (0.06–0.80)*	1.05 (0.60–1.83)	2.38 (1.06–5.34)*	1.13 (0.53–2.39)
Yes	296 (25.4)	**–**	**–**	**–**	**–**	**–**	**–**
Living in local lockdown							
Yes	79 (6.8)	0.68 (0.13–3.53)	1.67 (0.55–5.05)	4.86 (1.09–21.60)*	0.09 (0.01–0.61)*	1.05 (0.28–4.04)	3.19 (1.16–8.75)*
No	1087 (93.2)	**–**	**–**	**–**	**–**	**–**	**–**
Depression caseness							
Yes	257 (22.1)	0.86 (0.20–3.66)	2.07 (0.83–5.18)	1.61 (0.40–6.50)	2.20 (0.95–5.13)	0.89 (0.30–2.67)	1.52 (0.67–3.45)
No	909 (77.9)	**–**	**–**	**–**	**–**	**–**	**–**
GAD caseness							
Yes	205 (17.6)	2.03 (0.50–8.20)	2.06 (0.81–5.24)	2.07 (0.54–8.00)	0.84 (0.32–2.15)	1.32 (0.40–4.39)	1.22 (0.51–2.92)
No	961 (82.4)	**–**	**–**	**–**	**–**	**–**	**–**
C-19 PTSD caseness							
Yes	185 (15.8)	0.94 (0.29–3.06)	1.98 (0.89–4.41)	5.88 (1.70–20.41)**	1.85 (0.84–4.07)	0.92 (0.29–2.91)	2.20 (1.02–4.74)*
No	981 (84.2)	**–**	**–**	**–**	**–**	**–**	**–**
C-19 suicidal ideation							
Yes	160 (15.2)	1.31 (0.45–3.88)	0.89 (0.40–1.94)	4.24 (1.22–14.71)*	0.92 (0.40–2.11)	1.24 (0.50–3.08)	3.26 (1.55–6.85)**
No	890 (84.8)	**–**	**–**	**–**	**–**	**–**	**–**
Psychological variables							
Loneliness	5.00 (3–9)	1.04 (0.80–1.34)	1.22 (1.01–1.48)*	1.05 (0.72–1.53)	1.08 (0.90–1.29)	1.21 (0.98–1.50)	1.49 (1.21–1.83)***
Hopefulness	6.42 (2–10)	1.55 (1.19–2.01)***	0.92 (0.77–1.09)	0.90 (0.64–1.28)	1.06 (0.91–1.25)	1.27 (1.04–1.55)*	1.17 (0.96–1.41)
Happiness	4.37 (1–7)	1.51 (0.94–2.43)	1.21 (0.87–1.70)	0.65 (0.33–1.27)	1.43 (1.08–1.89)*	1.16 (0.82–1.63)	0.82 (0.59–1.15)
Social support	26.27 (8–40)	1.01 (0.97–1.05)	0.98 (0.95–1.01)	0.98 (0.92–1.04)	1.03 (1.01–1.06)*	1.01 (0.98–1.05)	0.98 (0.95–1.01)
Resilience	19.68 (6–30)	0.91 (0.82–1.01)	0.99 (0.91–1.08)	1.12 (0.95–1.33)	1.01 (0.95–1.08)	1.04 (0.96–1.13)	1.04 (0.96–1.13)
Death anxiety	43.19 (17–85)	1.05 (1.01–1.08)*	1.00 (0.97–1.02)	1.07 (1.02–1.13)**	1.00 (0.99–1.02)	0.99 (0.96–1.01)	1.01 (0.99–1.04)
Intolerance of uncertainty	35.32 (12–60)	0.98 (0.93–1.04)	1.02 (0.98–1.06)	0.97 (0.90–1.05)	1.02 (0.98–1.05)	1.04 (1.00–1.09)	0.98 (0.94–1.03)

Class 6 (no difference) is reference class. Significant ORs in bold

*p < 0.05

**p < 0.01

***p < 0.001

The better relationships class (Class 4) were more likely to have had a 2019 household income of £1112 + per week (OR = 3.17) and £301–£490 per week (OR = 3.17), were more likely to be in a relationship (OR = 4.75), and were less likely to have a family member with a chronic health condition (OR = 2.08) and to live in a local lockdown area (OR = 11.11). These individuals also had greater levels of happiness (OR = 1.43) and social support (OR = 1.03). A better lifestyle with the exception of partner relationships (Class 5) was associated with being younger (OR = 0.97), being economically active (OR = 3.26), not being in a relationship (OR = 4.17), having lost income as a result of the pandemic (OR = 1.99), not being a keyworker (OR = 2.38) and having higher levels of hopefulness (OR = 1.27). Finally, worse partner relationship only (Class 7) was associated with having a 2019 income of £741–£1,111 (OR = 3.18) and £491–£740 (OR = 3.84), being in a relationship (OR = 5.48), having dependent children in the home (OR = 2.75), having lost income as a result of the pandemic (OR = 2.06), living in a smaller home (2 bedroom compared to 5 bedroom; OR = 13.79), living in a local lockdown (OR = 3.19), having a family member with a health condition (OR = 2.18), having higher levels of loneliness (OR = 1.49), meeting the criteria for COVID-19 PTSD (OR = 2.20) and reporting suicidal ideation since the beginning of the pandemic (OR = 3.26).

## Discussion

The seven classes identified in the current analyses revealed a number of interesting things about the lifestyle of the UK adult population 4 months after the first nationwide lockdown. These can be summarised as three key findings. First, the largest class, which comprised over two-thirds of the sample, reported virtually no change across the five lifestyle dimensions. The size of this class suggests that over the first 4 months of the pandemic, the quality of most individuals’ lifestyles remained largely unchanged compared to their pre-pandemic lives. Second, nearly one-in-six individuals in the sample reported that they had experienced improvements in two or more areas of their lifestyle compared to before the pandemic (Classes 1, 4 and 5). Overall lifestyle improvement, improvement in partner and family and friend relations only, and better lifestyle with the exception of partner relationship, were generally characterised by a unique pattern of sociodemographic, COVID-19 and psychological associations. Moreover, none of these classes were associated with any of the four mental health covariates. Third, Classes 2, 3, and 7 comprised nearly one-in-seven individuals in the sample, and represented those members of the UK population whose lifestyle had worsened in relation to at least one dimension of pandemic-related lifestyle change.

Class 3 exhibited the most extreme deterioration in lifestyle change and this was evident across all dimensions. Compared to Class 3, Class 2 was characterised by a similar but less severe pattern of negative change across all dimensions except the partner relations dimension, where little change was identified. Conversely, Class 7 exhibited relatively little change across all lifestyle dimensions, except partner relations, where the level of deterioration was similar to that identified in Class 3. Overall, therefore, class composition suggested that, when lifestyle quality changed during the pandemic, it centred around either positive/negative changes (i) across all aspects of lifestyle quality, (ii) across all areas except partner relationships and (iii) to partner relationships only (or in the case of Class 4, positive changes to the quality of relationships generally).

Of the negative lifestyle change classes, compared to the no-change baseline majority (i.e. Class 6), only Classes 3 and 7 were more likely to experience mental health problems. Members of both classes were more likely to meet caseness for COVID-19 traumatic stress and report suicidal ideation. Members of both classes were also more likely to be in a relationship, and live in an area that was under local lockdown. Classes 3 and 7 were, however, distinct from each other in relation to other covariates. Membership of Class 3 (deterioration across all dimensions) was uniquely predicted by city living, being white, an absence of an underlying health condition, and being a keyworker, while membership of Class 7 (deterioration in partner relations only) was predicted by a medium income level, children in the home, living in a two-bedroom property, lost income due to the pandemic, the presence of an underlying health condition, and higher levels of loneliness. Somewhat surprisingly, while Class 2 experienced deterioration in four of the five lifestyle dimensions, it was not at greater risk of mental ill health and was predicted only by economic activity status and loneliness.

Class 3 clearly demonstrates that some members of the population are experiencing extreme difficulties during the pandemic and that multiple aspects of their lives are being affected. Their city living and frontline worker status are likely to impact their lifestyles and contribute to fears of contagion, infection, and illness that commonly arise from higher rates of transmission in more populated areas [51], and greater exposure in frontline working environments [52]. Class 7 suggests that compromised partner relations may be a particularly meaningful aspect of lifestyle change during the pandemic that, in itself, signals other important difficulties in household circumstances such as having dependent children in the home, living in a smaller house, higher levels of loneliness, having a family member with a chronic health condition (who may or may not live in the home) and having lost income, all factors known to compromise relationships and well-being pre-pandemic [53–57].

Compromised partner relations may also, however, be consequential to other pandemic related factors. A number of COVID-19-related studies have begun to explore this. Using data from an online nationally representative probability survey of 1,010 American adults in April 2020, for example, Luetke et al. [13] found that 34% of respondents in a relationship reported some degree of conflict with their romantic partners due to the spread of COVID-19 and its related restrictions. In addition, they reported that individuals who experienced frequent coronavirus-related conflict with their partner were significantly more likely to report decreased frequency of intimate and sexual behaviours compared to those not experiencing any such conflict, exhibiting a dose–response trend among partnered sexual behaviours. Pietromonaco et al. [30] suggest that facing COVID-19-related external stress is likely to increase harmful dyadic processes (e.g. hostility, withdrawal, less responsive support), which may undermine couples’ relationship quality. Moreover, these harmful effects are likely to be exacerbated by the broader pre-existing context in which couples’ relationships are situated (e.g. social class, minority status, age), and their individual vulnerabilities (e.g. attachment insecurity, depression). Prime, Wade and Browne [58] state that the COVID-19 pandemic poses an acute threat to the well-being of families due to challenges related to social disruption such as financial insecurity, caregiving burden, and confinement-related stress (e.g. crowding, changes to routine). According to these authors, the consequences of these difficulties are likely to be longstanding, in part because of the ways in which contextual risk permeates the structures and processes of family systems.

Finally, a growing number of studies have reported increases in domestic and intimate partner violence during the pandemic [59–61]. For example, a 60% increase in emergency calls from women subjected to violence by their intimate partner has been reported in the World Health Organization Europe member states. Comparing April 2020 with the same period last year, WHO reported that online inquiries to violence prevention support hotlines had also increased as much as fivefold [14]. Overall therefore, relationship complications (that may have existed before and/or been caused/compounded by the pandemic), alongside negative changes in other lifestyle dimensions in some cases, seem to constitute the more severe pandemic-related lifestyle quality change due to their association with poor mental health (COVID-19 PTSD and recent suicidal ideation) and are therefore an important marker for investigation.

The current study’s strengths include its sample size and use of weighting procedures to ensure the sample was nationally representative of the UK population across a number of sociodemographic indicators. Additionally, the sophisticated analytic strategy employed allowed for a better understanding of the complex ways in which the pandemic has affected people’s lives. There are, however, several limitations of the study to consider. Firstly, the cross-sectional design limits interpretations relating to causality. Secondly, the lifestyle indicator items asked participants to retrospectively reflect on their lives now compared to before the pandemic, and thus may be susceptible to recall bias. The current study used data from Wave 3 of the C19PRC Study, which was gathered during July 2020. At this time, the UK was past the peak of its first wave of coronavirus and the daily number of cases and deaths had reduced. As a result, lockdown measures were eased, and some individuals were able to engage with more ‘normal’ routines. Future research should consider pandemic-related lifestyle change in the light of further waves of coronavirus cases and the reinstatement of lockdown periods. Third, quota sampling was used to recruit participants via non-probability, opt-in online survey panels which excluded participants who did not have access to the Internet and those who could not read or write in English. Relatedly, this sampling strategy may have been susceptible to a number of biases (sampling bias, non-response bias, demand characteristics bias, question order bias, personality biases, psychometric biases) that have been shown to undermine confidence in online survey research findings [62–64]. Fourth, the low number of cases in some of the latent classes in the best fitting LPA model may have impeded the detection of some effects. Finally, although framed around the pandemic, we do not know with certainty whether these changes were as a result of the pandemic, either directly or indirectly.

## Supplementary Information

Below is the link to the electronic supplementary material.

Supplementary Online Resource 1 Supplementary methodological information (DOCX 45 KB)

## Data Availability

Survey data from the C19PRC Study UK Waves 1–3 can be found at https://osf.io/v2zur/files/.
