# Supplementary material for: Modelling the complexity of pandemic-related lifestyle quality change and mental health: an analysis of a nationally representative UK general population sample
Source: Soc Psychiatry Psychiatr Epidemiol. 2021 Dec 16;57(6):1247–60. doi: 10.1007/s00127-021-02210-w (PMC8674524; doi:10.1007/s00127-021-02210-w)
Supplement: Supplementary file 1 — Supplementary Online Resource 1 Supplementary methodological information (DOCX 45 KB) [file 127_2021_2210_MOESM1_ESM.docx]

**Supplementary material**

Online supplementary material for ‘Modelling the complexity of pandemic-related lifestyle quality change and mental health: An analysis of a nationally representative UK general population sample’ (Butter et al., 2021) submitted to Social Psychiatry and Psychiatric Epidemiology. Corresponding author: Dr Sarah Butter, [s.butter@sheffield.ac.uk](mailto:s.butter@sheffield.ac.uk)

- 1. **Details of predictor variables**

A series of predictor variables were extracted from Wave 1 (W1, March 2020) and Wave 3 (W3, July 2020) as follows:

Sociodemographic

Demographic variables of *age,* *gender* (male=1; female=2). Both were measured at W3.

*Urbanicity*: Participants were asked “Do you consider yourself to live in:” and were required to choose one of the options provided: ‘City’, ‘Suburb’, ‘Town’, or ‘Rural’. The variable was recoded to a binary variable representing urbanicity (1= City; 0 = Suburb, Town, or Rural). Urbanicity was measured at W1.

*Ethnicity:* Ethnicity was recoded into a binary variable (0= White British/Irish; 1 = White non-British/Irish; Indian, Pakistani, Chinese, Afro-Caribbean, African, Arab, Bangladeshi, Other Asian, or Other ethnic group). Ethnicity was measured at W1.

*Income:* Participants were asked “Please choose from the following options to indicate your approximate gross (before tax is taken away) household income in 2019 (last year). Include income from partners and other family members living with you and all kinds of earnings including salaries and benefits” to choose one of 5 categories: “£0 - £300 per week (equals about £0 - £1290 per month or £0 - 15,490 per year)”, “£301 - £490 per week (equals about £1,291 - £2,110 per month or £15,491 - £25,340 per year)”, “£491 - £740 per week (equals about £2,111 - £3,230 per month or £25,341 - £38,740 per year)”, “£741 - £1,111 per week (equals about £3,231 - £4,830 per month or £38,741 - £57,930 per year)”, and “£1,112 or more per week (equals about £4,831 or more per month or £57,931 or more per year)”. Gross household income was measured at W1.

*Economic activity:* Participants were asked to select if they were Employed full time, Employed part time (regular hours), Self-employed full time, Self-employed part time, Zero hours contract, Other flexible work practice, Been placed on the Government furlough scheme, Unemployed (because of coronavirus), Unemployed (not because of coronavirus), Full time student, Retired. These were recoded as binary variable with the first 7 options labelled ‘Economically active’ (0) and the last 4 options labelled ‘Economically inactive’ (1). Economic activity was measured at W3.

*Relationship status:* Participants were asked what their legal marital or same-sex civil status was. Responses options were ‘Married’, ‘Cohabiting’, ‘In a registered same-sex civil partnership’, ‘In a relationship but not living together’ (which were categorised as ‘In a relationship’ [0]), and ‘Never married and never registered same-sex civil partnership (single)’ ‘Separated, but still legally married’, ‘Divorced’, ‘Widowed’, ‘Separated but still legally in a same-sex partnership’, ‘Formally in a same-sex legal partnership which is now legally dissolved’, ‘Surviving partner from a same-sex civil partnership’ (which were considered ‘Not in a relationship’ [1]). Relationship status was measured at W3.

*Presence of dependent children in the home:* Participants were asked to report How many children (below the age of 18) lived in their household. Responses ranged from 0 through 10 or more. Endorsement of zero children or if the respondent previously reported living alone (and therefore were screened out of this question), were coded as ‘No children in the home’ (1). Reported one or more children was coded as ‘Children in the home’ (0). Presence of children in the home was measured at W3.

*Bedrooms in the household*: Participants were asked to specify how many bedrooms were in their home. Response options ranged from ‘None (I live in a single-room dwelling place e.g. studio apartment)’ to ‘5 or more’. Responses of ‘none’ and ‘1 bedroom’ were collapsed together for the purposes of the analysis. Number of bedrooms in the home was measured at W3.

COVID-19 related

*Loss of income:* Participants were asked “Please estimate the percentage change (either increase or decrease) in your monthly household income compared to the average monthly income before the COVID-19 pandemic.” They were presented with a sliding scale ranging from -100 (100% less) to 100 (100% greater or more), with 0 representing ‘no change’. Scores of -1 to -100 were categorised as lost income (0), scores of 0 to 100 were categorised as ‘no lost income (1). Lost income was measured at W3.

*Chronic illness self:* Participants were asked “Do you have diabetes, lung disease, or heart disease?” and the response options were ‘Yes’ (0) and ‘No’ (1). Chronic illness self was measured at W1.

*Chronic illness family*: They were also asked “Do any of your immediate family have diabetes, lung disease, or heart disease?” and the response options were ‘Yes’ (0) and ‘No’ (1). Chronic illness family was measured at W1.

*COVID-19 infection status, self:* An individual was considered to have ‘perceived COVID-19 infection’ (0) if they responded ‘yes’ to the question “To the best of your knowledge, to date, have you been infected by COVID-19?”, or they responded ‘I’m not sure’ and subsequently clarified their uncertainty status by endorsing the following response “I have had symptoms and I think I may have been infected with the COVID-19 virus, however I have not been tested.” Individuals who responded that they did not think they had been infected, or were unsure but endorsed either “I have had symptoms but I do not think I have been infected with the COVID-19 virus” or “I have not had symptoms but I still think I have been infected with the COVID-19 virus” responses were considered to have not been infected with COVID-19 (1). COVID-19 infection status (self) was measured at W3.

*COVID-19 infection status, family members:* An individual was considered to have a family member with perceived COVID-19 infection (0) if they responded ‘yes’ to the item “Has anyone else from your household been diagnosed with COVID-19 (confirmed by test)?” or “Has anyone from your extended family been diagnosed with COVID-19 (confirmed by test)?”. Responses of either ‘no’ or ‘not applicable’ to both of these items was categorised as not having a family member who has/had COVID-19 (1). COVID-19 infection status, family members was measured at W3.

*Key worker status*: Participants were provided with the definition of being a key worker as follows: “Key workers are people whose jobs are vital to public health and safety during the pandemic. Take a look at the list below and let us know if you are a key worker (tick the appropriate option).” Response options comprised “Health & social care worker (e.g. all NHS staff including administrative and cleaning staff, care home workers)”, “Education and child care (e.g. nursery care workers and teachers)”, “Food and other necessary goods (e.g. staff involved in production, processing, distribution, sale and delivery of goods)”, “Key public services (e.g. postal workers, those required to run the justice system, religious staff, those responsible for managing the deceased and journalists providing public service broadcasting)”, “Local and national government (e.g. staff in administrative roles essential to the effective delivery of the COVID-19 response or delivering essential public services including payment of benefits)”, “Utility workers (e.g. staff needed to keep oil, gas, electricity, water and sewerage operations running, staff in the civil nuclear, chemical, and telecom communication sectors)”, “Public safety and national security (e.g. police and support staff, Ministry of Defence civilian staff and armed forces personnel, fire and rescue staff and workers responsible for border security, prisons and probation)”, and “Transport (e.g. staff keeping air, water, road and rail passenger and freight transport modes operating)”. Endorsement of any o these options was categorised as being a key worker (1). Additionally, participants could respond “I am not a keyworker” (0). The key worker item was not asked to participants who previously endorsed being unemployed (not because of coronavirus), a fulltime student or retired. These individuals were categorised as not being a key worker. Key worker status was measured at W3.

*Living in an area under local lockdown*: Participants were given the following definition of a ‘local lockdown’ and asked if they lived in an area that was under local lockdown at the time they were completing the survey: “A local lockdown is a partial or full re-introduction of measures to control the spread of the coronavirus in a specific locality. The aim of a local lockdown is to control the spread of the coronavirus pandemic by containing it within a particular area and so avoid re-imposing social distancing restrictions across the whole of the country. On 29 June, Matt Hancock, the health secretary, announced that the first local lockdown would be applied in Leicester.” Responses were coded ‘Yes’ (1) and ‘No’ (2). Living in an area under local lockdown was measured at Wave 3.

*COVID-19 related anxiety*: The survey included a question “How anxious are you about the coronavirus COVID-19 pandemic?” and the participants were provided with a ‘slider’ (electronic visual analogue scale) to indicate their degree of anxiety with ‘0’ and ‘100’ at the left and right hand extremes respectively, and 10 point increments. This produced continuous scores ranging from 0 to 100 with higher scores reflecting higher levels of COVID-19 related anxiety. The scores were recoded into ‘Low’ (0 - 33), ‘Moderate’ (34 - 67), and ‘High’ (68 - 100). COVID-19 related anxiety was measured at W3.

*Close COVID-19 death:* Participants were asked: “Has anyone close to you died because of COVID-19?” This was collapsed into a binary variable categorised as ‘Yes/Unsure (not certain COVID-19 was the cause of death)’ (0) and ‘No’ (1). Close COVID-19 death was measured at W3.

Psychological variables

*Loneliness*: The three-item Loneliness Scale [1] asks participants to indicate how often they feel they lack companionship, left out, and isolated from others. Responses are scored using a three-point scale including (1) ‘Hardly Ever’, (2) ‘Sometimes’, and (3) ‘Often’. Possible scores range from 3 to 9 with higher scores indicating higher levels of loneliness. The internal reliability of the scale scores in this sample was excellent (*α* = .88). Loneliness emasured at W3.

*Resilience:* The Brief Resilience Scale (BRS) [2] comprises six-items answered using a five-point Likert scale ranging from ‘Strongly Disagree’ (1) to ‘Strongly Agree’ (5). Possible scores range from 6 to 30 with higher scores indicating higher levels of resilience. The internal reliability in this sample was excellent (*α* = .89). Resilience measured at W1.

*Death anxiety*: The Death Anxiety Inventory (DAI) [3] includes 17 items based on a five-point Likert scale ranging from (1) ‘Totally Disagree’ to (5) ‘Totally Agree’. Higher scores indicate higher levels of death anxiety. The internal reliability of the DAI scores in this sample was excellent (*α* = .94). Death anxiety measured at W1.

*Intolerance of uncertainty*: The Intolerance of Uncertainty scale (IUS) [4] includes 12 items (answered using a five-point Likert scale ranging from (1) ‘Not at All Characteristic of Me’ to (5) ‘Entirely Characteristic of Me’ (5). Higher scores indicate increased levels of intolerance of uncertainty. The internal reliability of the IUS scores in this sample was excellent (*α* = .90). Intoelrance of Uncertainty measured at W1.

*Hopefulness:* Respondents were asked to complete the Brief-H-Pos Scale [5], a two-item measure which is a positive re-framing of the negatively worded two-item Hopeless Scale [6]. The two items are scored on a 5-point Likert scale ranging from 1, absolutely disagree to 5, absolutely agree (higher scores indicate higher levels of hopefulness). The internal reliability of hopefulness scores was excellent in this sample (*α* = .84). Hopefulness measured at W3.

*Happiness:* The Subjective Happiness Scale (SHS) [7] is a 4-item self-report measure of self-reported happiness. Respondents are to consider a series of statements and report on a 7-point Likert scale (ranging from 1, Not at all to 7, A great deal). A single composite score is computed by averaging the responses to the four items (fourth item is reverse coded) and higher scores reflect higher levels of happiness. The internal reliability of happiness scores was excellent in this sample (*α* = .83). Happiness measured at W3.

*Social support:* The Modified Medical Outcome Social Support Survey (mMOS-SSS) [8]. The 8-item mMOS-SSS, an abbreviated version of the 19-item MOS-SSS [9], ask respondents to report how frequently they have available to them two domains of social support (instrumental/tangible and emotional). Items are scored on a 5-point Likert scale ranging from 1, none of the time to 5, all of the time. The measure had excellent internal reliability in the current sample (*α* = .97). Social support measured at W3.

*Depression*

Nine symptoms of depression were measured using the Patient Health Questionnaire-9 (PHQ-9) [10]. Participants indicated how often they had been bothered by each symptom over the past 2 weeks using a four-point Likert scale ranging from 0 (not at all) to 3 (nearly every day). Possible scores ranged from 0 to 27, with higher scores indicative of higher levels of depression. To identify participants likely to meet the criteria for depressive disorder, a cut-off score of ≥10 was used. This cut-off produces adequate sensitivity (0.85) and specificity (0.89), corresponds to ‘moderate’ levels of depression [11] and is used to identify a level of depression that may require psychological intervention [12]. The psychometric properties of the PHQ-9 scores have been widely supported [13], and the reliability of the scale among the current sample was excellent (α = 0.93).

#### Generalised anxiety

Symptoms of generalised anxiety were measured using the Generalized Anxiety Disorder 7-item Scale (GAD-7) [14]. Participants indicated how often they had been bothered by each symptom over the past 2 weeks on a four-point Likert scale (0 = Not at all, to 3 = Nearly every day). Possible scores ranged from 0 to 21, with higher scores indicative of higher levels of anxiety. A cut-off score of 10 was used; this has been shown to result in sensitivity of 89 % and a specificity of 82 % [14]. The GAD-7 has been shown to produce reliable and valid scores in community studies [15], and the reliability in the current sample was excellent (α = 0.96).

*COVID-19 related posttraumatic stress*

The International Trauma Questionnaire [16] is a self-report measure of ICD-11 posttraumatic stress (PTSD) based on a total of six symptoms across the three symptom clusters of Re-experiencing, Avoidance, and Sense of Threat: each symptom cluster comprises two items. Participants were asked to complete the ITQ “in relation to [their] experience of the COVID-19 pandemic…[and] how much [they] have been bothered by that problem in the past month”. The PTSD symptoms are accompanied by three items measuring functional impairment caused by these symptoms. All items are answered on a 5-point Likert scale, ranging from 0 (Not at all) to 4 (Extremely) with possible PTSD scores ranging from 0–24. A score of ≥ 2 (Moderately) is considered ‘endorsement’ of that symptom. A PTSD diagnosis requires traumatic exposure, and at least one symptom to be endorsed from each PTSD symptom cluster (Re-experiencing, Avoidance, and Sense of Threat), and endorsement of at least one indicator of functional impairment. The psychometric properties of the ITQ scores have been demonstrated in multiple general population [17], and clinical and high-risk samples [18]. The reliability of the PTSD items in the current sample was excellent (α = 0.94).

*Suicidal ideation*

Following the PHQ-9, respondents were asked a series of items related to self-harm and suicide. This section was optional; participants who did not wish to take part in this section (*n=*99, 8.5%) were automatically skipped to the next survey measure. Participants were asked: *The COVID-19 pandemic represents a difficult time in many people’s lives when they may become very miserable and depressed and may feel like taking drastic action because of these feelings. Since the pandemic began, have you ever thought of harming yourself or taking your life, even if you would not really do it?* Participants responded either ‘Yes’ (1) or ‘No’ (0).

**Table 1 OS** Correlations among pandemic-related lifestyle change indicators

|  | 1 | 2 | 3 | 4 | 5 | 6 | 7 | 8 | 9 | 10 | 11 | 12 | 13 | 14 | 15 | 16 | 17 | 18 |
| --- | --- | --- | --- | --- | --- | --- | --- | --- | --- | --- | --- | --- | --- | --- | --- | --- | --- | --- |
| 1. Home life |  |  |  |  |  |  |  |  |  |  |  |  |  |  |  |  |  |  |
| 2. Partner | **.536** |  |  |  |  |  |  |  |  |  |  |  |  |  |  |  |  |  |
| 3. Family | **.347** | **.290** |  |  |  |  |  |  |  |  |  |  |  |  |  |  |  |  |
| 4. Children | **.270** | **.288** | **.410** |  |  |  |  |  |  |  |  |  |  |  |  |  |  |  |
| 5. Diet | **.233** | **.140** | **.119** | **.095** |  |  |  |  |  |  |  |  |  |  |  |  |  |  |
| 6. Exercise | **.191** | **.193** | **.158** | **.114** | **.474** |  |  |  |  |  |  |  |  |  |  |  |  |  |
| 7. Mental health | **.317** | **.270** | **.128** | **.109** | **.352** | **.326** |  |  |  |  |  |  |  |  |  |  |  |  |
| 8. Physical health | **.315** | **.209** | **.200** | **.147** | **.455** | **.675** | **.474** |  |  |  |  |  |  |  |  |  |  |  |
| 9. Work-life balance | **.318** | **.216** | **.180** | **.096** | **.213** | **.159** | **.315** | **.260** |  |  |  |  |  |  |  |  |  |  |
| 10. Work role | **.281** | **.195** | **.129** | **.096** | **.202** | **.169** | **.277** | **.239** | **.483** |  |  |  |  |  |  |  |  |  |
| 11. Colleagues | **.236** | **.196** | **.094** | **.118** | **.187** | **.111** | **.216** | **.183** | **.299** | **.441** |  |  |  |  |  |  |  |  |
| 12. Commuting | **.211** | **.183** | **.128** | **.149** | **.097** | **.120** | **.153** | **.116** | **.319** | **.252** | **.205** |  |  |  |  |  |  |  |
| 13. Education | **.291** | **.249** | **.152** | **.113** | **.156** | **.153** | **.308** | **.193** | **.321** | **.265** | **.337** | **.305** |  |  |  |  |  |  |
| 14. Socialising | **.140** | **.110** | **.086** | .031 | **.185** | **.148** | **.237** | **.211** | **.132** | **.231** | **.174** | .003 | **.223** |  |  |  |  |  |
| 15. Hobbies | **.264** | **.241** | **.251** | **.183** | **.126** | **.214** | **.303** | **.270** | **.241** | **.202** | **.195** | **.218** | **.375** | **.223** |  |  |  |  |
| 16. Religious | **.187** | **.177** | **.196** | **.127** | **.187** | **.162** | **.212** | **.184** | **.191** | **.175** | **.182** | **.192** | **.378** | **.216** | **.309** |  |  |  |
| 17. Social media | **.148** | **.103** | **.124** | **.064** | .032 | .044 | **.113** | **.107** | **.139** | **.137** | **.198** | **.108** | **.242** | **.077** | **.199** | **.191** |  |  |
| 18. Friends | **.237** | **.144** | **.269** | **.203** | **.140** | **.146** | **.208** | **.198** | **.207** | **.205** | **.253** | **.074** | **.218** | **.248** | **.248** | **.171** | **.164** |  |
| 19. Sex life | **.236** | **.444** | **.213** | **.222** | **.124** | **.178** | **.253** | **.215** | **.206** | **.178** | **.204** | **.137** | **.243** | **.250** | **.282** | **.212** | **.127** | **.173** |

Significant correlations (*p* <0.05) in bold

**References**

1 Hughes ME, Waite LJ, Hawkley LC, Cacioppo JT (2004) A short scale for measuring loneliness in large surveys: Results from two population-based studies. Res Aging 26: 655-72.

2 Smith BW, Dalen J, Wiggins K, et al. (2008) The brief resilience scale: assessing the ability to bounce back. Int J Behav Med 15: 194-200.

3 Tomás-Sábado J, Gómez-Benito J, Limonero JT (2005) The death anxiety inventory: A revision. Psychol Rep 97: 793-6.

4 Buhr K, Dugas MJ (2002) The intolerance of uncertainty scale: Psychometric properties of the English version. Behav Res Ther 40: 931-45.

5 Fraser L, Burnell M, Salter LC, et al. (2014) Identifying hopelessness in population research: a validation study of two brief measures of hopelessness. BMJ Open 4.

6 Everson SA, Goldberg DE, Kaplan GA, et al. (1996) Hopelessness and risk of mortality and incidence of myocardial infarction and cancer. Psychosom Med 58: 113-21.

7 Lyubomirsky S, Lepper HS (1999) A measure of subjective happiness: Preliminary reliability and construct validation. Soc Indic Res 46: 137-55.

8 Moser A, Stuck AE, Silliman RA, Ganz PA, Clough-Gorr KM (2012) The eight-item modified Medical Outcomes Study Social Support Survey: psychometric evaluation showed excellent performance. J Clin Epidemiol 65: 1107-16.

9 Sherbourne CD, Stewart AL (1991) The MOS social support survey. Soc Sci Med 32: 705-14.

10 Kroenke K, Spitzer RL, Williams JB (2001) The PHQ‐9: Validity of a brief depression severity measure. J Gen Intern Med 16: 606-13. doi:10.1046/j.1525-1497.2001.016009606.x.

11 Manea L, Gilbody S, McMillan D (2012) Optimal cut-off score for diagnosing depression with the Patient Health Questionnaire (PHQ-9): a meta-analysis. CMAJ 184: E191-E6.

12 National Collaborating Centre for Mental Health (2018) The improving access to psychological therapies manual. NHS. Available at <https://www.england.nhs.uk/publication/the-improving-access-to-psychological-therapies-manual/>

13 Kroenke K, Spitzer RL, Williams JB, Löwe B (2010) The patient health questionnaire somatic, anxiety, and depressive symptom scales: A systematic review. Gen Hosp Psychiatry 32: 345-59.

14 Spitzer RL, Kroenke K, Williams JB, Löwe B (2006) A brief measure for assessing generalized anxiety disorder: the GAD-7. Arch Intern Med 166: 1092-7. doi:10.1001/archinte.166.10.1092.

15 Hinz A, Klein AM, Brähler E, et al. (2017) Psychometric evaluation of the Generalized Anxiety Disorder Screener GAD-7, based on a large German general population sample. J Affect Disord 210: 338-44.

16 Cloitre M, Shevlin M, Brewin CR, et al. (2018) The International Trauma Questionnaire: development of a self‐report measure of ICD‐11 PTSD and complex PTSD. Acta Psychiatr Scand 138: 536-46. doi:10.1111/acps.12956.

17 Ben‐Ezra M, Karatzias T, Hyland P, et al. (2018) Posttraumatic stress disorder (PTSD) and complex PTSD (CPTSD) as per ICD‐11 proposals: A population study in Israel. Depress Anxiety 35: 264-74.

18 Hyland P, Shevlin M, Brewin CR, et al. (2017) Validation of post‐traumatic stress disorder (PTSD) and complex PTSD using the International Trauma Questionnaire. Acta Psychiatr Scand 136: 313-22.
